# Supplementary material for: EnzML: multi-label prediction of enzyme classes using InterPro signatures
Source: BMC Bioinformatics. 2012 Apr 25;13:61. doi: 10.1186/1471-2105-13-61 (PMC3483700; doi:10.1186/1471-2105-13-61)
Supplement: Addtional file 5 — The Java code to format the data files, evaluate and predict. The file enzml_java_code.tar.gz contains the Java code used to format database data to ARFF and XML formats, to execute cross and train-test (jackknife) evaluations and to record evaluation results to database. More information is included in the readme.txt file and the Javadoc files. The code can be used with a MySQL database. To use a different database software, other JDBC drivers might be required. [file 1471-2105-13-61-S5.gz › java_code/enzml2011/doc/uk/ac/ed/inf/taweka/class-use/Initialisation.html]

Uses of Interface uk.ac.ed.inf.taweka.Initialisation


---


|  |  |  |
| --- | --- | --- |
| **Overview** \| **Package** \| **Class** \| **Use** \| **Tree** \| **Deprecated** \| **Index** \| **Help** | |  |
| PREV   NEXT | **FRAMES**    **NO FRAMES**     **All Classes** |  |


---


## **Uses of Interface uk.ac.ed.inf.taweka.Initialisation**

| Packages that use Initialisation | |
| --- | --- |
| **uk.ac.ed.inf.taweka** |  |
| **uk.ac.ed.inf.taweka.mulan** |  |

Uses of Initialisation in uk.ac.ed.inf.taweka

| Classes in uk.ac.ed.inf.taweka that implement Initialisation | |
| --- | --- |
| `class` | `ArffGenerator`             Creates an ARFF file: checks file properties, loads data from database, fills and writes to file the ARFF attributes, classes and instances, generates auxiliary files if needed. |
| `class` | `ArffProperties`             Contains the properties for a Weka ARFF file It contains: 1. |

Uses of Initialisation in uk.ac.ed.inf.taweka.mulan

| Classes in uk.ac.ed.inf.taweka.mulan that implement Initialisation | |
| --- | --- |
| `class` | `MulanArffGenerator`             Generates an Attribute Relation Format File (ARFF) in Mulan format (multiple class labels per instance). |
| `class` | `MulanArffProperties`             Class |

---


|  |  |  |
| --- | --- | --- |
| **Overview** \| **Package** \| **Class** \| **Use** \| **Tree** \| **Deprecated** \| **Index** \| **Help** | |  |
| PREV   NEXT | **FRAMES**    **NO FRAMES**     **All Classes** |  |


---
